# Mediation Mendelian randomization analysis of immune cell phenotypes and glioma risk: unveiling the regulation of cerebrospinal fluid metabolites

**DOI:** 10.1007/s12672-025-02499-y

**Published:** 2025-05-09

**Authors:** Siyuan Zhao, Jinghao Jiang, Jianwu Zhang, Xiaoqing Jin

**Affiliations:** 1 Emergency Center, Zhongnan Hospital of Wuhan University, 169 Donghu Road, Wuchang, Wuhan, 430071 Hubei China; 2 The Second Clinical College, Wuhan University, Wuhan, 430071 Hubei China; 3 Emergency Department, Guilin People’s Hospital, 12 Wenming Road, Xiangshan, Guilin, 541000 Guangxi China; 4 Department of Laboratory, Zhongnan Hospital of Wuhan University, 169 Donghu Road, Wuhan, 430071 Hubei China

**Keywords:** Glioma, Immune cell phenotypes, Cerebrospinal fluid metabolites, Mediation Mendelian Randomization, Immune evasion

## Abstract

**Background and objective:**

Gliomas, particularly glioblastoma multiforme (GBM), are the most common primary central nervous system tumors in adults and are notoriously difficult to treat due to their high heterogeneity and invasiveness. Despite advances in molecular diagnostics and personalized therapies, prognosis remains poor. The immune system plays a critical role in glioma progression. This study employed mediation Mendelian randomization analysis to explore the relationships between immune cell phenotypes, cerebrospinal fluid metabolites, and glioma, aiming to uncover potential mechanisms of tumor progression and immune evasion.

**Method:**

In this study, we employed several analytical methods including IVW, MR Egger, Simple mode, Weighted median, and Weighted mode, with IVW results being considered the primary basis. We assessed heterogeneity and pleiotropy, and used leave-one-out analysis to determine sensitivity, ensuring the stability and reliability of the results. The potential mediating effects of cerebrospinal fluid metabolites were investigated to explore the underlying mechanisms linking immune cell function and glioma. The GWAS data for immune cells, cerebrospinal fluid metabolites, and glioma used in this study were sourced from public databases.

**Result:**

We identified nine risk immune cell phenotypes for glioma (such as CD19 on IgD( +) CD24(-)), and ten protective immune cell phenotypes (such as CD11c on monocytes). Mediation analysis revealed that levels of 7-alpha-hydroxy-3-oxo-4-cholestenoate (7-hoca) (MP = − 14.6%) and Palmitoyl dihydrosphingomyelin (d18:0/16:0) (MP = 7.9%) partially mediated the relationship between CD3 on CD39( +) resting Treg cells and glioma. Additionally, 7-hoca levels (MP = − 12.3%) and Phenyllactate (pla) levels (MP = 4.12%) partially mediated the association between FSC-A on NKT cells and glioma. Furthermore, Glycerophosphoinositol levels (MP = − 12.1%) and Orotate levels (MP = − 11.4%) partially mediated the relationship between Granulocyte adenylyl cyclase (Granulocyte AC) and glioma.

**Conclusion:**

This study identified that specific immune cell phenotypes directly influence glioma risk and indirectly modulate this risk through cerebrospinal fluid metabolites. CD19 on IgD( +) CD24(−) B cells were identified as risk factors, while CD11c on monocytes were protective. Metabolites like 7-hoca and glycerophosphoinositol play key mediating roles. These findings enhance our understanding of glioma pathophysiology and suggest that immune modulation and metabolic intervention may be promising therapeutic strategies.

**Supplementary Information:**

The online version contains supplementary material available at 10.1007/s12672-025-02499-y.

## Introduction

Glioblastoma multiforme (GBM), the most aggressive glioma subtype, accounts for nearly 50% of adult central nervous system malignancies [1–3]. Despite advancements in molecular diagnostics and multimodal therapies (e.g., surgery, radiotherapy), GBM remains incurable, with a median survival of less than 15 months [4–6]. This poor prognosis stems from tumor heterogeneity, invasive growth, and an immunosuppressive microenvironment dominated by regulatory T cells (Tregs) and myeloid-derived suppressor cells (MDSCs) [7–9].

The complexity of gliomas extends beyond the genetic and epigenetic alterations within tumor cells, encompassing the diverse cellular components and their interactions within the tumor microenvironment. In recent years, researchers have uncovered that the immune system plays a critical role in the pathogenesis and progression of gliomas. Immune cells within the tumor microenvironment, such as macrophages, T cells, and dendritic cells, engage in dynamic interactions with glioma cells, profoundly influencing tumor progression [8–10]. Immunosuppressive mechanisms within the tumor microenvironment, such as the upregulation of immune checkpoint molecules, enable tumor cells to evade host immune surveillance, thereby facilitating immune evasion [11, 12].

The immunosuppressive glioma microenvironment is characterized by infiltrating Tregs and MDSCs, which secrete cytokines (e.g., TGF-β, IL-10) and metabolites (e.g., NO) to inhibit effector T and NK cell activity [6, 7, 13–15]. These mechanisms facilitate tumor immune evasion and resistance to conventional therapies.

Recent advances in cerebrospinal fluid metabolomics have highlighted its utility in identifying prognostic biomarkers for neurological malignancies, underscoring the rationale for our focus on metabolite-mediated pathways [16]. The upregulation of immune checkpoint molecules is particularly crucial in the immune evasion mechanisms of gliomas. The expression of immune checkpoint molecules such as programmed death-ligand 1 (PD-L1) and cytotoxic T-lymphocyte-associated protein 4 (CTLA-4) is markedly increased in gliomas, inhibiting the activity of effector T cells and inducing the expansion of Tregs [17, 18]. These immunosuppressive molecules transmit negative signals by binding to their corresponding receptors, leading to the functional inhibition and apoptosis of T cells. This mechanism has been recognized as a major reason for the poor responsiveness of glioma patients to immunotherapy.

Metabolic reprogramming further sustains glioma progression. The Warburg effect generates lactate, acidifying the microenvironment and impairing dendritic cell activity [19–21]. Simultaneously, tryptophan catabolism via IDO/TDO enzymes depletes local tryptophan and produces kynurenine, promoting Treg expansion and T cell dysfunction [22]. These metabolic adaptations synergize with immune suppression to drive tumor immune evasion. Tryptophan metabolism, particularly through the kynurenine pathway, is highly active in gliomas. Enzymes such as indoleamine 2,3-dioxygenase (IDO) and tryptophan 2,3-dioxygenase (TDO) catalyze the breakdown of tryptophan, leading to the production of metabolites like kynurenine, which suppress T cell function and promote the expansion of Tregs [22]. This contributes to the immunosuppressive microenvironment characteristic of gliomas, enabling the tumor to evade immune detection and response​.

Mendelian randomization (MR) analysis is a method that uses genetic variants as instrumental variables to assess the causal relationship between exposures and outcomes. It effectively reduces the impact of confounding factors and has been widely applied in epidemiological studies in recent years [23]. This study aims to explore the relationship between gliomas, immune cells, and cerebrospinal fluid metabolites using a mediation MR approach, thereby revealing potential mechanisms involved in tumor initiation, progression, and immune evasion. This research not only deepens our understanding of the pathobiology of gliomas but may also identify new therapeutic targets for future interventions.

## Method

### Design

**Fig. 1:**
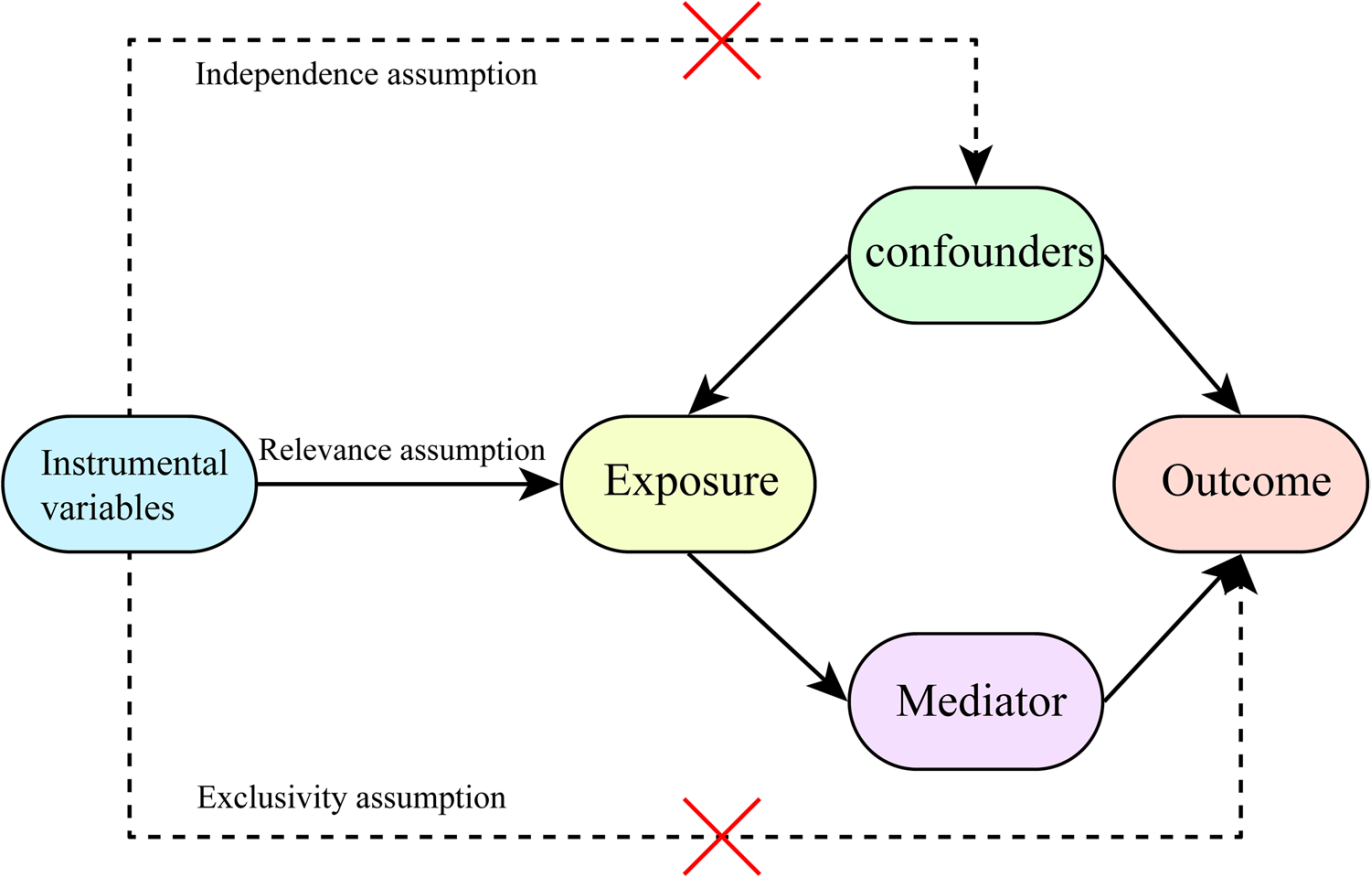
Three fundamental assumptions of Mendelian randomization (MR) analysis

We investigated the causal relationship between 731 immune cell phenotypes and glioma using Mendelian randomization analysis, simultaneously exploring whether cerebrospinal fluid metabolites mediate this relationship. We used single nucleotide polymorphisms (SNPs) as instrumental variables that met the following conditions: (1) The instrumental variable had to be strongly associated with the exposure; (2) The instrumental variable cannot be associated with any confounding factors; (3) The instrumental variables can affect the outcome through exposure, rather than by any other manner to affect the outcome (Fig. 1). MR analysis results including inverse variance weighted (IVW) [24], MR Egger [25], Simple mode [26] and Weighted median [27], weighted mode [26], including IVW as main results. In addition, Ethical approval and consent to participate in the original genome-wide association studies (GWASs) were obtained from relevant review boards. The current study used publicly available summary statistics data, and thus no additional ethics approval was required [28]. This study adheres to the Strengthening the Reporting of Observational Studies in Epidemiology using Mendelian Randomization (STROBE-MR) guidelines. A completed STROBE-MR checklist is provided as Supplementary File 10, detailing compliance with all 25 reporting items.

### Data sources

#### The GWAS data for glioma

GWAS data for glioma were obtained from FinnGen consortium (Risteys FinnGen R10—C3_GBM).The dataset counted 253 individuals, including 95 females and 158 males. The glioma GWAS data were obtained from the FinnGen consortium (R10 release), comprising 253 individuals (95 females, 158 males) of European ancestry diagnosed with glioblastoma or astrocytoma. Diagnoses were confirmed histologically according to WHO 2021 criteria. Genotyping was performed using the Illumina Global Screening Array, followed by imputation with the TOPMed reference panel. Quality control steps included: (1) SNP exclusion for call rate < 95%; (2) sample exclusion for heterozygosity outliers or sex mismatch; (3) Hardy–Weinberg equilibrium (HWE) testing (*P* > 1 × 10⁻⁶). Association analyses were conducted using logistic regression adjusted for age, sex, and the first 10 principal components.

#### The GWAS data for immune cell phenotypes

The GWAS data on immune cell phenotypes were obtained from a cohort study involving 3757 individuals of European ancestry (accession numbers from ebi-a-GCST0001391 to ebi-aGCST0002121) [29]. The immune cell phenotype GWAS data were derived from a European cohort of 3757 individuals (Orrù et al., 2020). Immune cell subsets (e.g., CD19 + B cells, CD11c + monocytes) were quantified via flow cytometry, and phenotypes were standardized as absolute counts or median fluorescence intensity (MFI). Genotyping used the Illumina Infinium Omni2.5–8 array, with imputation based on the 1000 Genomes Phase 3 reference. Quality control included SNP filtering (MAF > 1%, imputation accuracy R^2^ > 0.3) and sample exclusion for relatedness (PI-HAT > 0.2). Linear regression models adjusted for age, sex, and batch effects were applied for association testing.

#### The GWAS data for cerebrospinal fluid metabolites

A study utilizing a cohort of Alzheimer’s disease patients conducted a comprehensive metabolomic and genome-wide association analysis of 338 cerebrospinal fluid metabolites. This analysis identified 16 genotype-metabolite associations and ultimately delineated 19 significant cerebrospinal fluid metabolite-phenotype associations [30]. The GWAS data for cerebrospinal fluid (CSF) metabolites were derived from a cohort of Alzheimer’s disease (AD) patients due to the lack of publicly available metabolomic GWAS datasets specific to glioma or healthy populations. Although AD and glioma differ in etiology, they share overlapping metabolic pathways (e.g., cholesterol metabolism, neuroinflammation-related metabolites), making this dataset biologically plausible for exploratory analyses. To address potential cohort-specific biases, we conducted sensitivity analyses (e.g., MR-Egger intercept test) and confirmed that the selected SNPs exhibited no significant horizontal pleiotropy (*P* > 0.05). Furthermore, the AD cohort provided the largest CSF metabolome GWAS summary statistics with comprehensive metabolite coverage (n = 338), which was critical for detecting subtle mediation effects. The CSF metabolomic GWAS data were generated from a cohort of 1,203 Alzheimer’s disease patients (Panyard et al., 2021). Metabolite levels were quantified using liquid chromatography-mass spectrometry (LC–MS), covering 338 metabolites. Genotyping utilized the Illumina HumanOmni5Exome array, with imputation to the Haplotype Reference Consortium panel. Quality control involved metabolite normalization (log2-transformed and z-scored), SNP exclusion (MAF < 5%, imputation R^2^ < 0.6), and covariate adjustment for age, sex, and APOE ε4 status in linear models.

Detailed GWAS metadata, including phenotype definitions and access codes, are provided in Supplementary Table 9.

### IVs screening

We first selected instrumental variables strongly associated with the exposure based on the *P*-value, with a threshold set at *P* ≤ 5 × 10^–5^. Additionally, we removed variables with linkage disequilibrium, applying a threshold of r^2^ < 0.01 and kb > 10,000. We used the F-statistic to estimate the strength of instrumental variables. Instrumental variables with an F-value < 10 are considered weak IV and will bias the results significantly. We therefore retained IVs with F > 10. In cases where the target SNP was unavailable in the outcome dataset, we selected proxy SNPs in high linkage disequilibrium (LD) with the target SNP (r^2^ ≥ 0.8) using the 1000 Genomes European reference panel. Proxy SNPs were only included if they met the same significance threshold (*P* ≤ 5 × 10⁻^5^) and LD criteria (r^2^ < 0.01, kb > 10,000). Palindromic SNPs (e.g., A/T or C/G alleles) were excluded to avoid strand ambiguity. For SNPs with allele frequency differences between exposure and outcome datasets, we further aligned the strands using the 1000 Genomes reference panel to ensure consistent effect direction.

### The assessment of heterogeneity and horizontal pleiotropy

In MR analysis tool must ensure that the variable can only be through the exposure factor to influence the outcome, so we adopt MR–Egger intercept test to detect pleiotropy, which reflects the stability of the research. When the *P*-value of the MR-Egger intercept is less than 0.05, horizontal pleiotropy is deemed to be present; conversely, if the *P*-value is greater than 0.05, horizontal pleiotropy is considered to be absent [31]. Employing Cochran’s Q statistic along with its corresponding *P*-value provides a quantitative evaluation of the heterogeneity among the selected IVs [32]. Sensitivity analyses were performed using the leave-one-out method to determine the effect of individual SNPs on the results [33].

### Analysis of the overall causal effect

We use a bidirectional two-sample MR analysis to assess 731 types of immune cell phenotype with glioma. IVW was considered as the primary outcome, while the results of the other four analyses, such as MR Egger, were supplemented. To account for multiple testing across 731 immune cell phenotypes, we applied the Benjamini–Hochberg false discovery rate (FDR) correction. A significance threshold of FDR-adjusted P < 0.05 was used to define robust associations.

### Mediation analysis

The effects of immune cell phenotypes on glioma can be divided into direct effects and indirect effects mediated through cerebrospinal fluid metabolites. Here, we used a two-step MR approach to calculate the estimated causal effect of immune cell phenotypes on cerebrospinal fluid metabolites (beta1) and cerebrospinal fluid metabolites on glioma (beta2), and then we could calculate the mediating effect and the proportion of the mediating effect in the total effect.

## Results

### Total effect of immune cell phenotypes on glioma

To investigate the overall effect of immune cell phenotypes on glioma, we employed two-sample MR analysis, including IVW, MR Egger, Simple mode, Weighted median, and Weighted mode methods. Ultimately, we identified immune cell phenotypes from the IVW results with a *P*-value < 0.05, where the odds ratios (OR) were consistently greater than 1 or less than 1, and the pleiotropy *P*-value was > 0.05. Among these, 9 immune cell types, such as CD19 on IgD( +) CD24(−), were identified as risk factors for glioma (Supplementary Fig. 1), while 10 immune cell types, such as CD11c on monocyte, were considered protective factors for glioma (Supplementary Fig. 2). The summarized results of the MR analysis are provided in Supplementary Tables 1, 2.

In the screening process, a threshold of P > 0.05 was set for pleiotropy, ensuring that the significant immune cell phenotypes obtained do not exhibit SNP pleiotropy (Supplementary Table 3). The method used to detect pleiotropy was the MR-Egger intercept method. Additionally, the screening included conditions where the OR values were either greater than 1 or less than 1. Therefore, the results obtained were consistent across all five statistical analysis models used in the main MR analysis, which more strongly supports the effect of certain immune cell phenotypes on the disease.

To mitigate potential heterogeneity from other factors affecting the MR results, we employed both the IVW and MR-Egger methods to assess heterogeneity, with a significance threshold of *P* < 0.05 (Supplementary Table 4). The results indicated that all immune cell phenotypes obtained exhibited no significant heterogeneity under both the IVW and MR-Egger models, suggesting that the results are not biased by heterogeneity (Supplementary Fig. 3). We further validated the results through sensitivity analysis and found no evidence that any single SNP significantly influenced the overall causal relationship (Supplementary Fig. 4).

### The mediating role of cerebrospinal fluid metabolites in immune cell-glioma interactions

The exploration of cerebrospinal fluid metabolite mediation between immune cells and glioma aims to investigate the potential mechanisms through which immune cells influence glioma.

First, using MR analysis, we investigated the relationships between all cerebrospinal fluid metabolites and glioma. Based on the results from the IVW method, we identified ten significant cerebrospinal fluid metabolites, such as 7-alpha-hydroxy-3-oxo-4-cholestenoate (7-hoca) levels, using a threshold of *P* < 0.05 (Supplementary Fig. 5). Subsequently, applying the same approach with a threshold of *P* < 0.05 based on IVW results, we identified immune cell phenotypes that significantly affect these mediator factors (Supplementary Table 5). By comparing immune cell phenotypes causally associated with disease and those significantly associated with cerebrospinal fluid metabolites, we identified a set of nine immune cell-cerebrospinal fluid metabolite pairs (Supplementary Table 6).

Next, we investigated their mediating effects to determine whether the cerebrospinal fluid metabolite acts as a mediator. The study encompassed the mediation effect of immune cell phenotypes on cerebrospinal fluid metabolites (beta1) (Supplementary Table 7), as well as the mediation effect of cerebrospinal fluid metabolites on glioma (beta2) (Supplementary Table 8). Ultimately, we identified six meaningful pairs of immune cell-cerebrospinal fluid metabolite interactions.

**Fig. 2:**
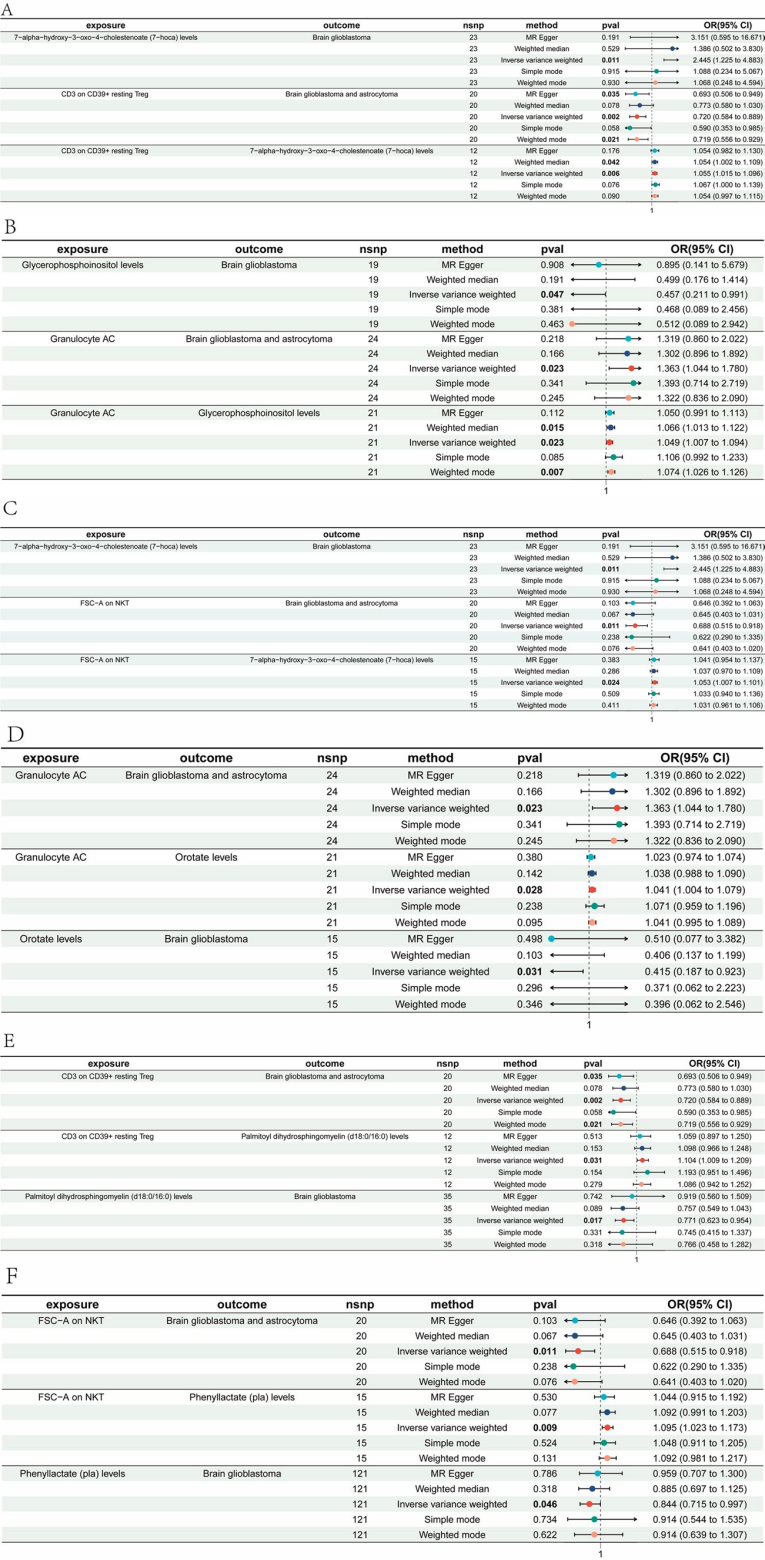
Forest plots for six significant immune cell-cerebrospinal fluid metabolite-glioma interactions. **A** CD3 on CD39( +) resting regulatory T cells (Tregs)–7-alpha-hydroxy-3-oxo-4-cholestenoate (7-hoca) levels–glioma. **B** Granulocyte adenylyl cyclase (Granulocyte AC)–Glycerophosphoinositol levels–glioma. **C** Forward scatter area (FSC-A) on natural killer T cells (NKT)–7-hoca levels–glioma. **D** Granulocyte AC–Orotate levels–glioma. **E** CD3 on CD39( +) resting Tregs–Palmitoyl dihydrosphingomyelin (d18:0/16:0) levels–glioma. **F** FSC-A on NKT–Phenyllactate (pla) levels–glioma

Ultimately, we found that CD3 on CD39(+) resting Tregs combined with 7-hoca levels jointly reduce the risk of glioma(Mediated effect, ME = 0.0479; Mediated proportion, MP = − 14.6%) (Table 1). FSC-A on NKT cells combined with 7-hoca levels decrease glioma risk(ME = 0.046, MP = − 12.3%) (Table 1). Granulocyte adenylyl cyclase (Granulocyte AC) combined with Glycerophosphoinositol levels jointly increase glioma risk(ME = − 0.0376, MP = − 12.1%) (Table 1), whereas Granulocyte AC combined with Orotate levels increase glioma risk(ME = − 0.0354, MP = − 11.4%) (Table 1). CD3 on CD39( +) resting Treg cells combined with Palmitoyl dihydrosphingomyelin (d18:0/16:0) levels jointly reduce glioma risk(ME = − 0.0259, MP = 7.9%) (Table 1). FSC-A on NKT cells combined with Phenyllactate (pla) levels decrease glioma risk (ME = − 0.0154, MP = 4.12%) (Table 1) (Fig. 2).

**Table 1:**
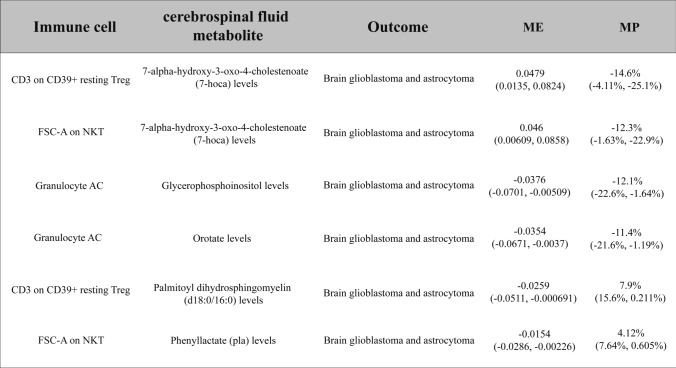
Six pairs of cerebrospinal fluid metabolites with “immune cell phenotype—Brain glioblastoma and astrocytoma” mediating effects

7-hoca, 7-alpha-hydroxy-3-oxo-4-cholestenoate; pla, Phenyllactate; AC, adenylyl cyclase; FSC-A, Forward scatter area; NKT, natural killer T cells; Tregs, regulatory T cells

## Discussion

This study uses mediation Mendelian randomization analysis to explore the complex relationships between immune cell phenotypes, cerebrospinal fluid metabolites, and glioma. The findings reveal that specific immune cell phenotypes not only directly influence glioma risk but also modulate this risk indirectly through the regulation of cerebrospinal fluid metabolites.

### Direct effects of immune cell phenotypes on glioma

The role of the immune system in tumorigenesis and tumor progression has been extensively studied, and this study further elucidates the direct impact of specific immune cells on glioma using MR analysis. Specifically, CD19 on IgD(+) CD24(−) B cells were identified as risk factors for glioma in this study. This cell subset is typically associated with B cell maturation and antigen presentation, suggesting that these cells may contribute to tumor growth and invasiveness by promoting inflammatory responses and altering the tumor microenvironment [34, 35]. Moreover, the abnormal activation of B cells could trigger a cascade of immune responses, such as antibody-dependent cellular cytotoxicity and complement-dependent cytotoxicity, which may further exacerbate tumor development [36].

Additionally, the hyperactivity of CD19(+) B cells may lead to autoimmune responses, which in some cases could promote tumor immune evasion, allowing tumor cells to escape immune surveillance. This is consistent with the observed associations between certain tumors and autoimmune diseases in some studies [37, 38]. Therefore, regulating B cell function, particularly through targeted interventions at specific subsets, could provide novel strategies for the prevention or treatment of glioma.

In contrast, the protective role of CD11c on monocytes suggests that certain immune cell subsets may inhibit tumor progression within the tumor microenvironment. CD11c(+) monocytes are precursor cells to dendritic cells, which play a critical role in activating and regulating immune responses. Research has shown that these cells can enhance anti-tumor immune responses by presenting antigens to T cells and activating NK cells, thereby inhibiting tumor growth [39]. Furthermore, CD11c(+) cells may promote Th1-type immune responses by secreting cytokines such as IL-12 and IFN-γ, which are typically associated with anti-tumor activity [40, 41].

These findings provide new insights into the role of the immune system in glioma, particularly in the balance between tumor immune evasion mechanisms and immune surveillance. Additionally, the study's application of multiple Mendelian randomization methods, such as IVW and MR-Egger analysis, rigorously excluded potential confounding factors like SNP pleiotropy and heterogeneity, further supporting the reliability of the results. Overall, specific immune cell phenotypes have direct and significant impacts on glioma, offering new clues for understanding the tumor's pathological mechanisms and providing clear directions for future research on immunotherapy.

### Mediating role of cerebrospinal fluid metabolites in immune cell-glioma interactions

In this study, we not only revealed the direct impact of immune cells on glioma but also delved into the critical role that cerebrospinal fluid metabolites play as mediators in this process. Changes in cerebrospinal fluid metabolites are considered direct reflections of the metabolic state of the central nervous system, and abnormalities in these metabolites are closely associated with the development of various neurological diseases.

The study identified several cerebrospinal fluid metabolites that are significantly associated with glioma, including 7-hoca. This metabolite is an intermediate product in the cholesterol metabolism pathway, and cholesterol plays a crucial role in cell membrane composition, signal transduction, and cell proliferation and survival [42, 43]. Disruptions in cholesterol metabolism are linked not only to cardiovascular diseases but also to the development of various cancers. Changes in 7-hoca levels may directly affect the survival environment of tumor cells by altering cell membrane rigidity, the distribution and function of membrane receptors, and intercellular signaling pathways, thereby influencing the risk of glioma [43–45].

More importantly, this study revealed interactions between specific immune cell phenotypes and cerebrospinal fluid metabolites. For example, the combination of CD3 on CD39(+) resting Tregs and 7-hoca levels significantly reduces glioma risk. This finding suggests that Tregs may indirectly influence tumor growth and progression by regulating metabolite levels in cerebrospinal fluid [18, 46, 47]. Tregs typically play a crucial role in suppressing excessive immune responses and maintaining immune tolerance. However, in the tumor environment, they may promote tumor growth by inhibiting anti-tumor immune responses [13, 48]. The interaction between Tregs and metabolites provides a new perspective for understanding the mechanisms of tumor immune evasion.

Additionally, the study found that the combination of granulocyte AC and glycerophosphoinositol increases glioma risk. Glycerophosphoinositol is a metabolite closely related to phospholipid metabolism, playing a key role in maintaining cell membrane structure and function, as well as in regulating various cell signaling pathways. In tumor cells, elevated glycerophosphoinositol levels may accelerate tumor occurrence and development by activating pro-tumor signaling pathways, promoting cell proliferation, and enhancing resistance to apoptosis [49]. The activation of Granulocyte AC is closely associated with inflammatory responses, indicating that inflammation and metabolic abnormalities jointly contribute to the pathogenesis of glioma [50].

These results highlight that cerebrospinal fluid metabolites are not only indicators of the metabolic state of the central nervous system but may also play a crucial mediating role in the impact of immune cells on glioma. Regulating the levels of these metabolites could offer new targets for early diagnosis and treatment of glioma. This finding also suggests directions for future research, such as exploring more interactions between metabolites and immune cells, and their roles in other central nervous system tumors. This will provide new insights into the comprehensive understanding of tumor pathogenesis and offer potential pathways for developing new therapeutic approaches.

This study highlights the significant role of specific immune cell phenotypes and cerebrospinal fluid metabolites in glioma, offering important implications for clinical practice. Firstly, these immune cell phenotypes and metabolites can serve as potential biomarkers for early screening and prognosis evaluation. For individuals identified as high-risk, such as those with elevated proportions of CD19 on IgD(+) CD24(−) B cells or abnormal levels of 7-hoca, early interventions like immunomodulators or metabolic interventions could potentially reduce the risk of glioma development.

Additionally, the interactions between immune cells and metabolites uncovered in this study suggest new therapeutic targets. Modulating the activity of CD3 on CD39(+) Tregs or regulating key metabolites in cerebrospinal fluid could influence the tumor microenvironment, thereby inhibiting tumor progression. This provides a scientific basis for developing personalized therapeutic strategies based on immune modulation and metabolic intervention.

Future research directions could expand in several areas. Firstly, further studies should validate these findings across different glioma subtypes and other central nervous system tumors to assess their broader applicability. Additionally, multicenter studies should be conducted to further verify the reliability and sensitivity of these immune cell phenotypes and metabolites as biomarkers. Secondly, research could delve deeper into the specific mechanisms by which these immune cell phenotypes and metabolites function within the tumor microenvironment, particularly in relation to immune evasion, inflammatory responses, and metabolic reprogramming. Lastly, clinical trials could evaluate the efficacy and safety of interventions based on these findings, offering new strategies for glioma treatment.

Despite the valuable insights provided by this study, there are several limitations that need to be acknowledged. Firstly, MR analysis relies on the selection of genetic instruments, which may be influenced by genetic variation and gene-environment interactions. While multiple statistical methods were used in this study to minimize these effects, the choice of instrumental variables may still affect the accuracy of the results.

Secondly, there may be heterogeneity in the sample sources of this study, particularly in its application to different populations, which could be limited. Differences in genetic background and environmental factors may impact the generalizability of the results across different racial or geographic populations. Future studies should include larger, more diverse population samples to enhance the external validity of the findings.

Third, the CSF metabolomic data were sourced from an AD cohort, which may introduce disease-specific confounding. However, we mitigated this concern by focusing on metabolites with known roles in both neurodegenerative and oncogenic pathways (e.g., 7-hoca in cholesterol metabolism). Future studies should validate these findings using glioma-specific metabolomic datasets once available.

Fourth, although we implemented FDR correction to mitigate false positives, the stringent threshold may have obscured subtle associations. Future studies with larger sample sizes are needed to validate these findings. Additionally, our exclusion of palindromic SNPs and reliance on proxy SNPs may have reduced instrument variable coverage, potentially omitting relevant genetic variants.

Fifth, the European ancestry of all GWAS datasets limits the generalizability of our findings to other populations. Future studies should prioritize multi-ethnic cohorts to assess trans-ethnic consistency.

Moreover, this study primarily focused on specific immune cell phenotypes and cerebrospinal fluid metabolites, leaving out other potentially important biomarkers. The pathogenesis of glioma is complex, involving multiple biological pathways and interactions, so future research should broaden the scope of analysis to include a wider range of biomarkers and signaling pathways for a more comprehensive understanding of glioma pathophysiology.

Lastly, although this study employed Mendelian randomization methods to reduce the impact of confounding factors, inherent limitations of this method still exist. Therefore, future research should incorporate longitudinal studies and experimental validation to further confirm and extend these findings.

## Supplementary Information

Additional file 1.

Additional file 2.

Additional file 3.

Additional file 4.

Additional file 5.

Additional file 6.

Additional file 7.

Additional file 8.

Additional file 9.

Additional file 10.

Additional file 11.

Additional file 12.

Additional file 13.

Additional file 14.

Additional file 15.

## Data Availability

GWAS data for glioma were obtained from FinnGen consortium (Risteys FinnGen R10—C3_GBM).The dataset counted 253 individuals, including 95 females and 158 males. The GWAS data on immune cell phenotypes were obtained from a cohort study involving 3,757 individuals of European ancestry (accession numbers from ebi-a-GCST0001391 to ebi-aGCST0002121). A study utilizing a cohort of Alzheimer's disease patients conducted a comprehensive metabolomic and genome-wide association analysis of 338 cerebrospinal fluid metabolites. This analysis identified 16 genotype-metabolite associations and ultimately delineated 19 significant cerebrospinal fluid metabolite-phenotype associations. The GWAS data on immune cell phenotypes were obtained from a cohort study involving 3,757 individuals of European ancestry (Accession numbers from ebi-a-GCST0001391 to ebi-aGCST0002121) [29]. A study utilizing a cohort of Alzheimer's disease patients conducted a comprehensive metabolomic and genome-wide association analysis of 338 cerebrospinal fluid metabolites. This analysis identified 16 genotype-metabolite associations and ultimately delineated 19 significant cerebrospinal fluid metabolite-phenotype associations [30].

## Abbreviations

GBM: Glioblastoma multiforme
7-hoca: 7-Alpha-hydroxy-3-oxo-4-cholestenoate
Tregs: Regulatory T cells
MDSCs: Myeloid-derived suppressor cells
NO: Nitric oxide
NK: Natural killer
PD-L1: Programmed death-ligand 1
CTLA-4: Cytotoxic T-lymphocyte-associated protein 4
IDO: Indoleamine 2,3-dioxygenase
TDO: Tryptophan 2,3-dioxygenase
MR: Mendelian randomization
SNPs: Single nucleotide polymorphisms
IVW: Inverse variance weighted
GWASs: Genome-wide association studies
OR: Odds ratios
Granulocyte AC: Granulocyte adenylyl cyclase
pla: Phenyllactate

## References

[1] Schaff LR, Mellinghoff IK. Glioblastoma and other primary brain malignancies in adults: a review. JAMA. 2023;329(7):574–87. 36809318 10.1001/jama.2023.0023 PMC11445779

[2] Antonelli M, Poliani PL. Adult type diffuse gliomas in the new 2021 WHO classification. Pathologica. 2022;114(6):397–409. 36534419 10.32074/1591-951X-823 PMC9763975

[3] Osborn AG, et al. The 2021 World health organization classification of tumors of the central nervous system: what neuroradiologists need to know. AJNR Am J Neuroradiol. 2022;43(7):928–37. 35710121 10.3174/ajnr.A7462 PMC9262075

[4] Johanns TM, et al. Integrating multisector molecular characterization into personalized peptide vaccine design for patients with newly diagnosed glioblastoma. Clin Cancer Res. 2024;30(13):2729–42. 38639919 10.1158/1078-0432.CCR-23-3077 PMC11215407

[5] White J, et al. The tumour microenvironment, treatment resistance and recurrence in glioblastoma. J Transl Med. 2024;22(1):540. 38844944 10.1186/s12967-024-05301-9 PMC11155041

[6] Rong L, Li N, Zhang Z. Emerging therapies for glioblastoma: current state and future directions. J Exp Clin Cancer Res. 2022;41(1):142. 35428347 10.1186/s13046-022-02349-7 PMC9013078

[7] Ghaznavi H, et al. New insights into targeted therapy of glioblastoma using smart nanoparticles. Cancer Cell Int. 2024;24(1):160. 38715021 10.1186/s12935-024-03331-3 PMC11077767

[8] Xu S, et al. Immunotherapy for glioma: current management and future application. Cancer Lett. 2020;476:1–12. 32044356 10.1016/j.canlet.2020.02.002

[9] Sharma P, et al. Tumor microenvironment in glioblastoma: current and emerging concepts. Neurooncol Adv. 2023;5(1):vdad009. 36968288 10.1093/noajnl/vdad009 PMC10034917

[10] Di Nunno V, et al. Tumor-associated microenvironment of adult gliomas: a review. Front Oncol. 2022;12: 891543. 35875065 10.3389/fonc.2022.891543 PMC9301282

[11] Li JW, et al. The interplay between metal ions and immune cells in glioma: pathways to immune escape. Discov Oncol. 2024;15(1):348. 39134820 10.1007/s12672-024-01229-0 PMC11319581

[12] Qi FL, et al. Reversal of the immunosuppressive tumor microenvironment by nanoparticle-based activation of immune-associated cells. Acta Pharmacol Sin. 2020;41(7):895–901. 32467568 10.1038/s41401-020-0423-5 PMC7470798

[13] Li C, et al. Regulatory T cells in tumor microenvironment: new mechanisms, potential therapeutic strategies and future prospects. Mol Cancer. 2020;19(1):116. 32680511 10.1186/s12943-020-01234-1 PMC7367382

[14] Mi Y, et al. The emerging role of myeloid-derived suppressor cells in the glioma immune suppressive microenvironment. Front Immunol. 2020;11:737. 32391020 10.3389/fimmu.2020.00737 PMC7193311

[15] Lu J, et al. Myeloid-derived suppressor cells in cancer: therapeutic targets to overcome tumor immune evasion. Exp Hematol Oncol. 2024;13(1):39. 38609997 10.1186/s40164-024-00505-7 PMC11010322

[16] Zhang HY, et al. Global research trends in immunotherapy for glioma: a comprehensive visualization and bibliometric analysis. Front Endocrinol (Lausanne). 2023;14:1273634. 37867521 10.3389/fendo.2023.1273634 PMC10585102

[17] Qi Y, et al. Immune checkpoint targeted therapy in glioma: status and hopes. Front Immunol. 2020;11: 578877. 33329549 10.3389/fimmu.2020.578877 PMC7729019

[18] Regmi M, et al. From glioma gloom to immune bloom: unveiling novel immunotherapeutic paradigms-a review. J Exp Clin Cancer Res. 2024;43(1):47. 38342925 10.1186/s13046-024-02973-5 PMC10860318

[19] Strickland M, Stoll EA. Metabolic reprogramming in glioma. Front Cell Dev Biol. 2017;5:43. 28491867 10.3389/fcell.2017.00043 PMC5405080

[20] Agnihotri S, Zadeh G. Metabolic reprogramming in glioblastoma: the influence of cancer metabolism on epigenetics and unanswered questions. Neuro Oncol. 2016;18(2):160–72. 26180081 10.1093/neuonc/nov125 PMC4724176

[21] Platten M, et al. Tryptophan metabolism as a common therapeutic target in cancer, neurodegeneration and beyond. Nat Rev Drug Discov. 2019;18(5):379–401. 30760888 10.1038/s41573-019-0016-5

[22] Xu Y, et al. Immunomodulatory effects of tryptophan metabolism in the glioma tumor microenvironment. Front Immunol. 2021;12: 730289. 34659216 10.3389/fimmu.2021.730289 PMC8517402

[23] Moodie E, le Cessie S. Instrumental variables analysis and mendelian randomization for causal inference. J Infect Dis. 2024. 10.1093/infdis/jiae357. 39104210 10.1093/infdis/jiae357 PMC11911776

[24] Burgess S, Butterworth A, Thompson SG. Mendelian randomization analysis with multiple genetic variants using summarized data. Genet Epidemiol. 2013;37(7):658–65. 24114802 10.1002/gepi.21758 PMC4377079

[25] Bowden J, Davey SG, Burgess S. Mendelian randomization with invalid instruments: effect estimation and bias detection through Egger regression. Int J Epidemiol. 2015;44(2):512–25. 26050253 10.1093/ije/dyv080 PMC4469799

[26] Hartwig FP, Davey SG, Bowden J. Robust inference in summary data Mendelian randomization via the zero modal pleiotropy assumption. Int J Epidemiol. 2017;46(6):1985–98. 29040600 10.1093/ije/dyx102 PMC5837715

[27] Bowden J, et al. Consistent estimation in Mendelian randomization with some invalid instruments using a weighted median estimator. Genet Epidemiol. 2016;40(4):304–14. 27061298 10.1002/gepi.21965 PMC4849733

[28] Sanderson E, et al. Mendelian randomization. Nat Rev Methods Primers. 2022;2:6. 37325194 10.1038/s43586-021-00092-5 PMC7614635

[29] Orru V, et al. Complex genetic signatures in immune cells underlie autoimmunity and inform therapy. Nat Genet. 2020;52(10):1036–45. 32929287 10.1038/s41588-020-0684-4 PMC8517961

[30] Panyard DJ, et al. Cerebrospinal fluid metabolomics identifies 19 brain-related phenotype associations. Commun Biol. 2021;4(1):63. 33437055 10.1038/s42003-020-01583-z PMC7803963

[31] Burgess S, Thompson SG. Interpreting findings from Mendelian randomization using the MR-Egger method. Eur J Epidemiol. 2017;32(5):377–89. 28527048 10.1007/s10654-017-0255-x PMC5506233

[32] Bowden J, et al. Quantifying, displaying and accounting for heterogeneity in the meta-analysis of RCTs using standard and generalised Q statistics. BMC Med Res Methodol. 2011;11:41. 21473747 10.1186/1471-2288-11-41 PMC3102034

[33] Higgins JP, Thompson SG. Quantifying heterogeneity in a meta-analysis. Stat Med. 2002;21(11):1539–58. 12111919 10.1002/sim.1186

[34] Huang S, et al. Checkpoint CD24 function on tumor and immunotherapy. Front Immunol. 2024;15:1367959. 38487533 10.3389/fimmu.2024.1367959 PMC10937401

[35] Dixit N, et al. Leukocyte function antigen-1, kindlin-3, and calcium flux orchestrate neutrophil recruitment during inflammation. J Immunol. 2012;189(12):5954–64. 23144497 10.4049/jimmunol.1201638 PMC3723394

[36] Hu J, et al. Glioblastoma immunotherapy targeting the innate immune checkpoint CD47-SIRPalpha axis. Front Immunol. 2020;11: 593219. 33329583 10.3389/fimmu.2020.593219 PMC7728717

[37] Imura Y, et al. CD19-targeted CAR regulatory T cells suppress B cell pathology without GvHD. JCI Insight. 2020;5(14): e136185. 32525846 10.1172/jci.insight.136185 PMC7453900

[38] Michaelson JS, Baeuerle PA. CD19-directed T cell-engaging antibodies for the treatment of autoimmune disease. J Exp Med. 2024;221(5): e20240499. 38587494 10.1084/jem.20240499 PMC11001598

[39] In H, et al. Identification of dendritic cell precursor from the CD11c(+) cells expressing high levels of MHC class II molecules in the culture of bone marrow with FLT3 ligand. Front Immunol. 2023;14:1179981. 38094300 10.3389/fimmu.2023.1179981 PMC10716454

[40] Naradikian MS, et al. Cutting edge: IL-4, IL-21, and IFN-gamma interact to govern T-bet and CD11c expression in TLR-activated B cells. J Immunol. 2016;197(4):1023–8. 27430719 10.4049/jimmunol.1600522 PMC4975960

[41] Oka N, et al. IL-12 regulates the expansion, phenotype, and function of murine NK cells activated by IL-15 and IL-18. Cancer Immunol Immunother. 2020;69(9):1699–712. 32333080 10.1007/s00262-020-02553-4 PMC11027610

[42] Saeed A, et al. 7alpha-hydroxy-3-oxo-4-cholestenoic acid in cerebrospinal fluid reflects the integrity of the blood-brain barrier. J Lipid Res. 2014;55(2):313–8. 24319290 10.1194/jlr.P044982 PMC3886670

[43] Tao R, et al. Comprehensive analysis of the clinical and biological significances of cholesterol metabolism in lower-grade gliomas. BMC Cancer. 2023;23(1):692. 37488496 10.1186/s12885-023-10897-0 PMC10364387

[44] Guo X, et al. Cholesterol metabolism and its implication in glioblastoma therapy. J Cancer. 2022;13(6):1745–57. 35399707 10.7150/jca.63609 PMC8990433

[45] Zhang H, et al. Brain-computer interfaces: the innovative key to unlocking neurological conditions. Int J Surg. 2024;110(9):5745–62. 39166947 10.1097/JS9.0000000000002022 PMC11392146

[46] Xia C, et al. CD39/CD73/A2AR pathway and cancer immunotherapy. Mol Cancer. 2023;22(1):44. 36859386 10.1186/s12943-023-01733-x PMC9979453

[47] Zhang H, et al. Unveiling the landscape of cytokine research in glioma immunotherapy: a scientometrics analysis. Front Pharmacol. 2023;14:1333124. 38259287 10.3389/fphar.2023.1333124 PMC10800575

[48] Qin D, et al. Targeting tumor-infiltrating tregs for improved antitumor responses. Front Immunol. 2024;15:1325946. 38500876 10.3389/fimmu.2024.1325946 PMC10944859

[49] Patrussi L, et al. The glycerophosphoinositols: from lipid metabolites to modulators of T-cell signaling. Front Immunol. 2013;4:213. 23908653 10.3389/fimmu.2013.00213 PMC3725514

[50] Sim J, et al. Dysregulation of inflammasome activation in glioma. Cell Commun Signal. 2023;21(1):239. 37723542 10.1186/s12964-023-01255-5 PMC10506313

