# Supplementary material for: Mediation Mendelian randomization analysis of immune cell phenotypes and glioma risk: unveiling the regulation of cerebrospinal fluid metabolites
Source: Discov Oncol. 2025 May 9;16:712. doi: 10.1007/s12672-025-02499-y (PMC12064550; doi:10.1007/s12672-025-02499-y)
Supplement: Supplementary file 1 — Additional file 1. [file 12672_2025_2499_MOESM1_ESM.docx]

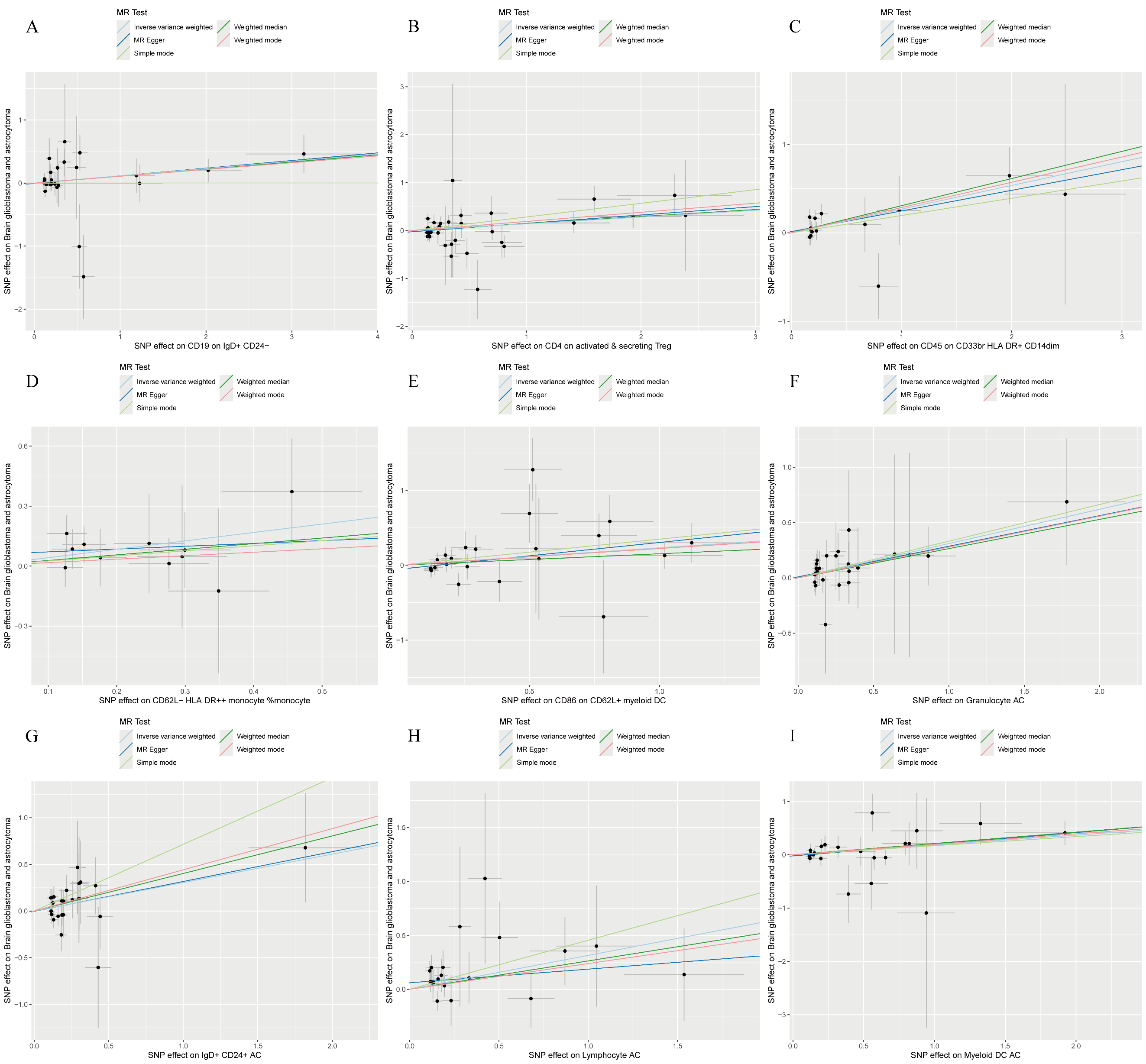


Supplementary Fig.1 9 immune cells was identified as potential risk factors for glioma basing on the results of the MR analysis. Among them, the results from the IVW method were used as the primary screening criterion, with a significance threshold set at P<0.05.

(A) CD19 on IgD+ CD24-, (B) CD4 on activated & secreting Treg, (C) CD45 on CD33br HLA DR+ CD14dim, (D) CD62L- HLA DR++ monocyte %monocyte, (E) CD86 on CD62L+ myeloid DC, (F) Granulocyte AC, (G) IgD+ CD24+ AC, (H) Lymphocyte AC, (I) Myeloid DC AC.
