# Supplementary material for: Mediation Mendelian randomization analysis of immune cell phenotypes and glioma risk: unveiling the regulation of cerebrospinal fluid metabolites
Source: Discov Oncol. 2025 May 9;16:712. doi: 10.1007/s12672-025-02499-y (PMC12064550; doi:10.1007/s12672-025-02499-y)
Supplement: Supplementary file 2 — Additional file 2. [file 12672_2025_2499_MOESM2_ESM.docx]

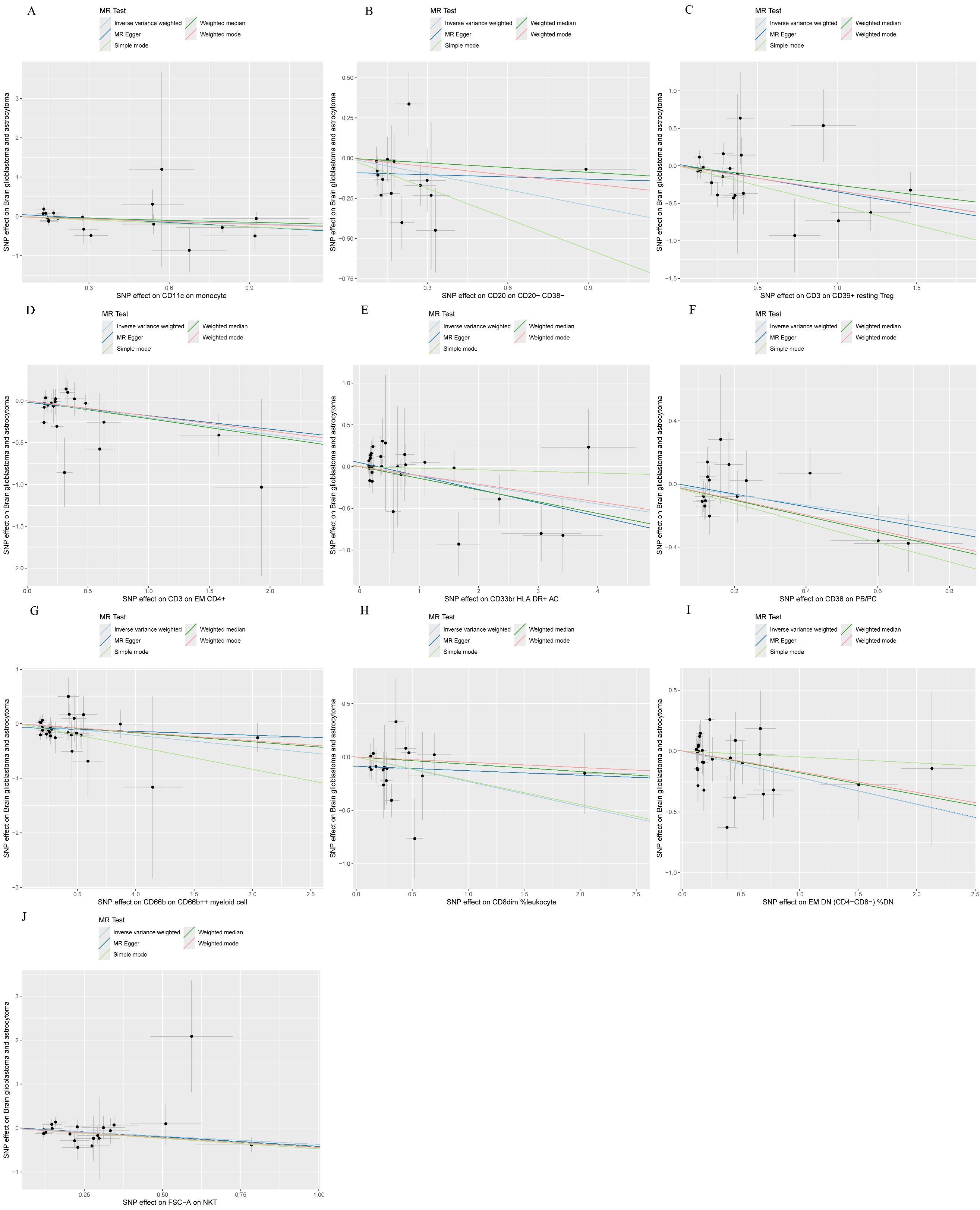
 Supplementary Fig.2 10 immune cells was identified as potential protective factors for glioma basing on the results of the MR analysis. Among them, the results from the IVW method were used as the primary screening criterion, with a significance threshold set at P<0.05.

(A) CD11c on monocyte, (B) CD20 on CD20- CD38-, (C) CD3 on CD39+ resting Treg, (D) CD3 on EM CD4+, (E) CD33br HLA DR+ AC, (F) CD38 on PB/PC, (G) CD66b on CD66b++ myeloid cell, (H) CD8dim %leukocyte, (I) EM DN (CD4-CD8-) %DN, (J) FSC-A on NKT.
