# Supplementary material for: Mediation Mendelian randomization analysis of immune cell phenotypes and glioma risk: unveiling the regulation of cerebrospinal fluid metabolites
Source: Discov Oncol. 2025 May 9;16:712. doi: 10.1007/s12672-025-02499-y (PMC12064550; doi:10.1007/s12672-025-02499-y)
Supplement: Supplementary file 3 — Additional file 3. [file 12672_2025_2499_MOESM3_ESM.docx]

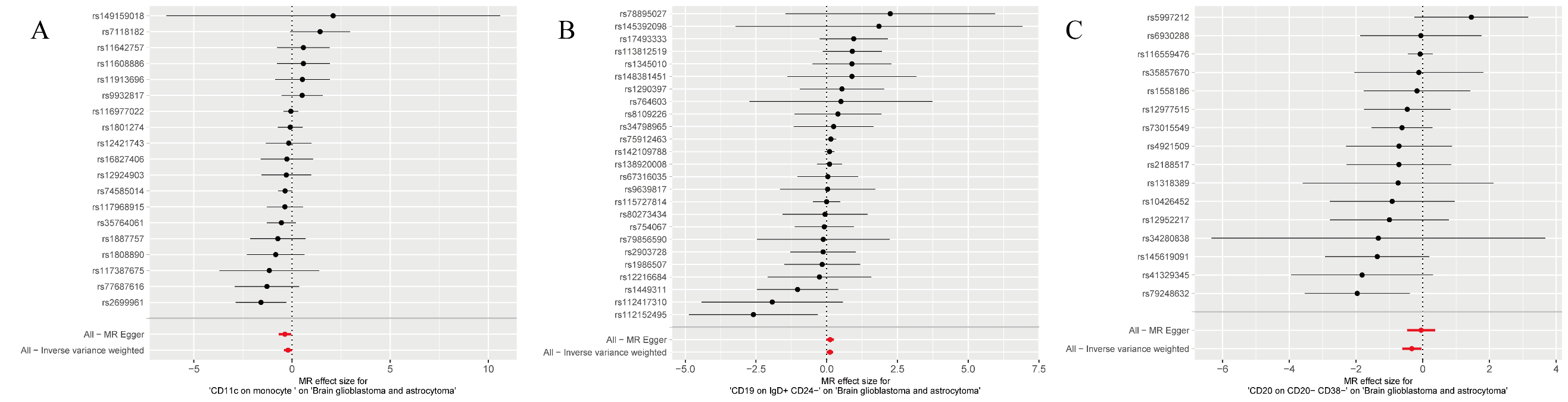

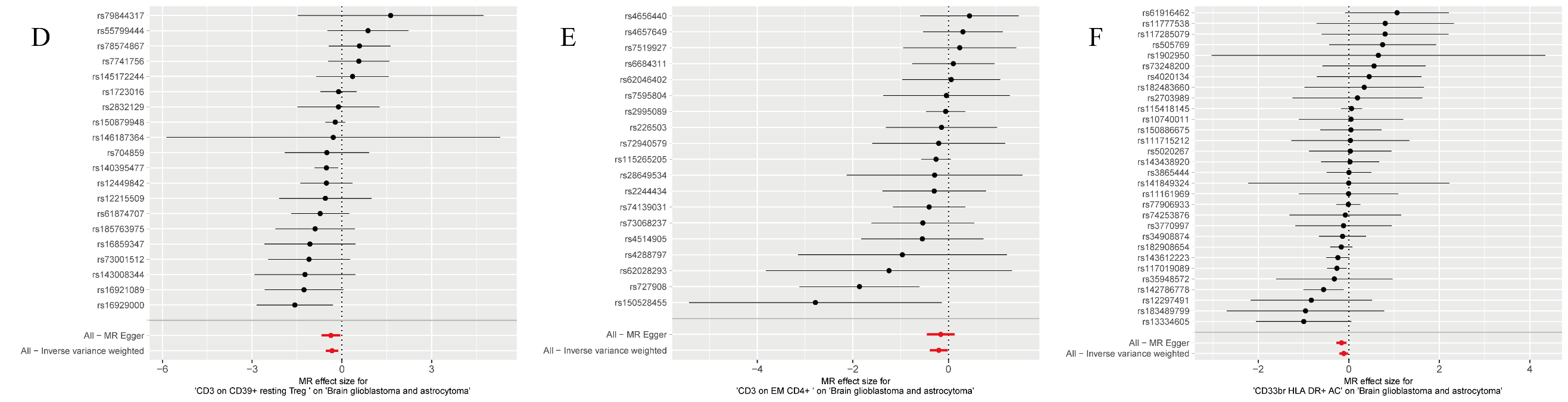

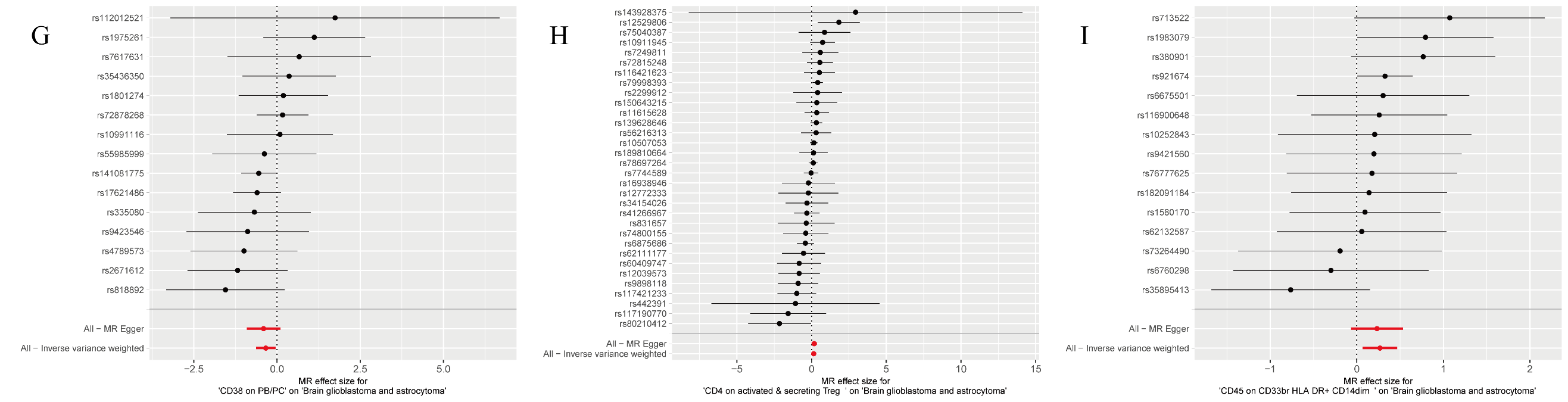

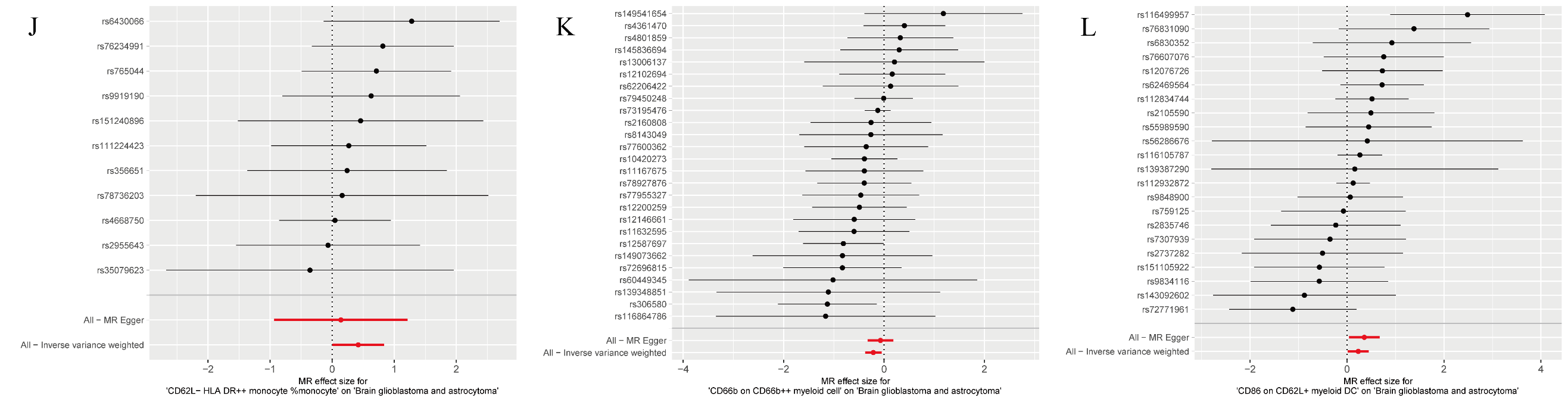

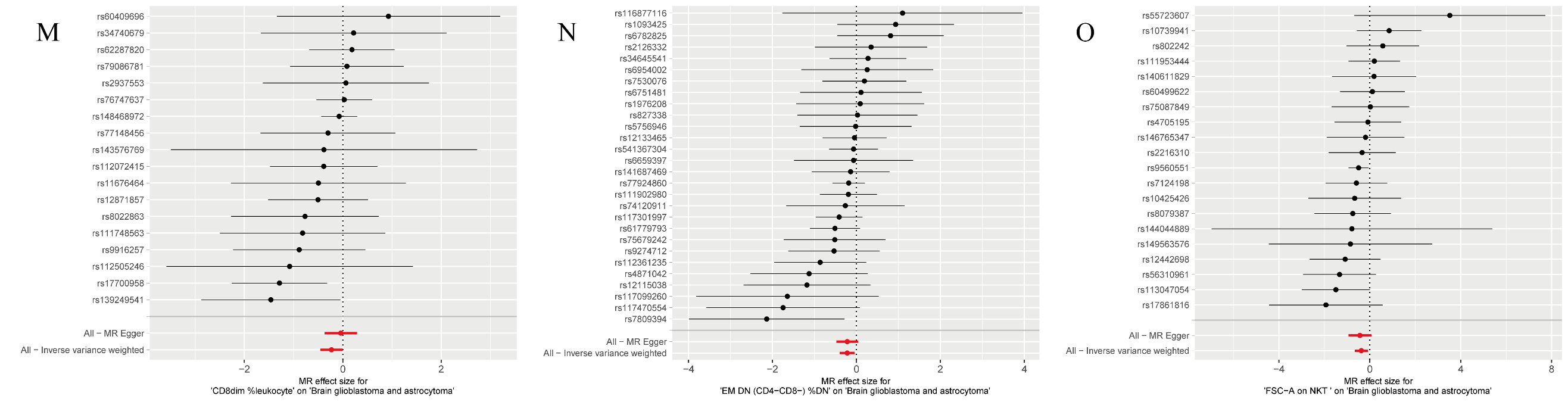

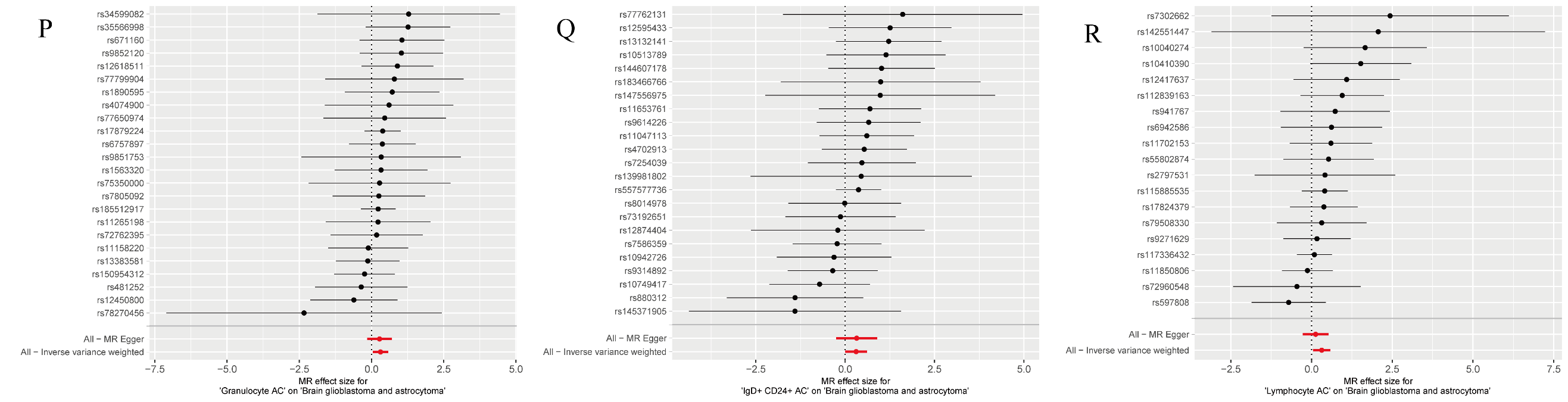

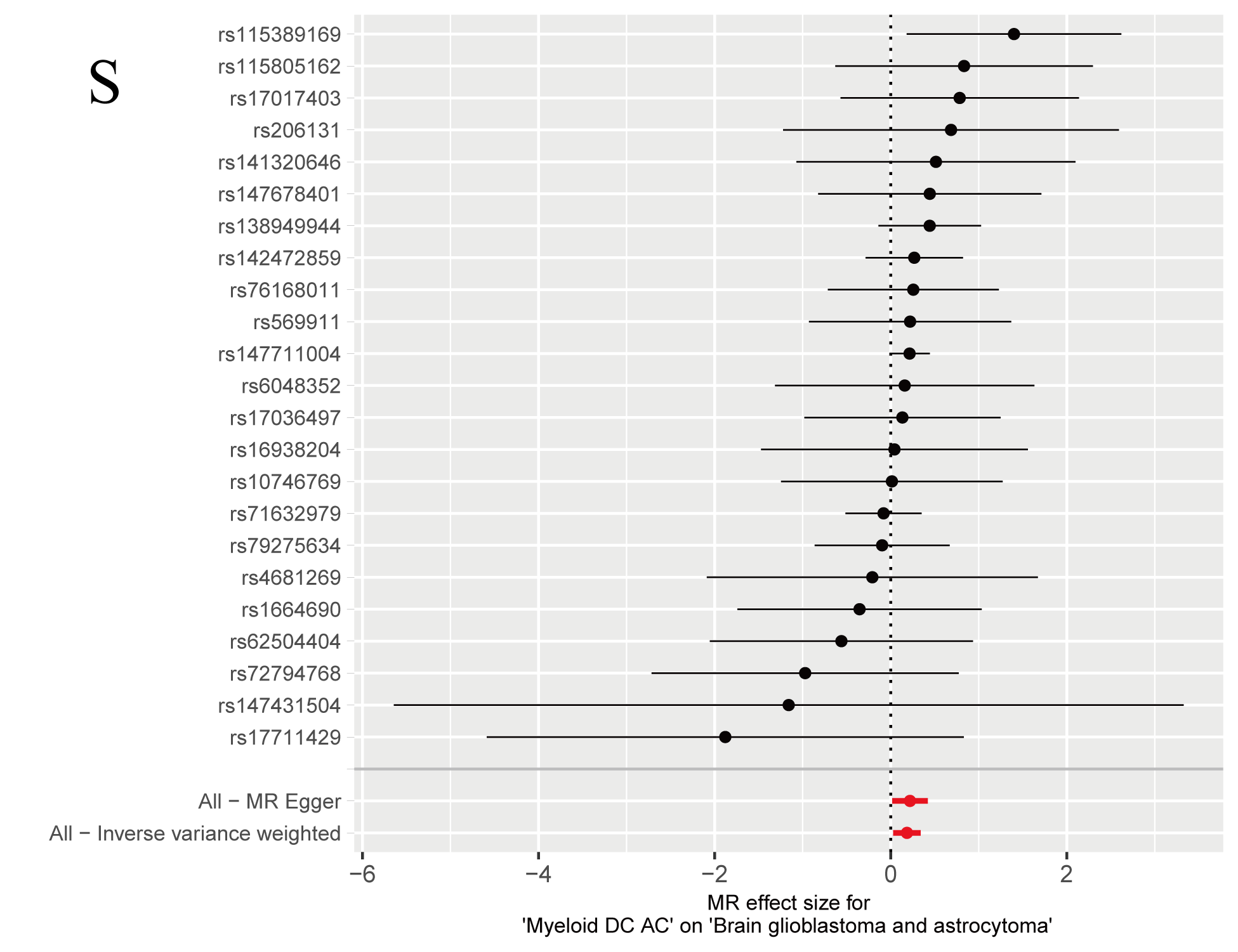


Supplementary Fig.3 The results indicated that all immune cell phenotypes obtained exhibited no significant heterogeneity under both the IVW and MR-Egger models, suggesting that the results are not biased by heterogeneity.
