# Supplementary material for: Mediation Mendelian randomization analysis of immune cell phenotypes and glioma risk: unveiling the regulation of cerebrospinal fluid metabolites
Source: Discov Oncol. 2025 May 9;16:712. doi: 10.1007/s12672-025-02499-y (PMC12064550; doi:10.1007/s12672-025-02499-y)
Supplement: Supplementary file 4 — Additional file 4. [file 12672_2025_2499_MOESM4_ESM.docx]

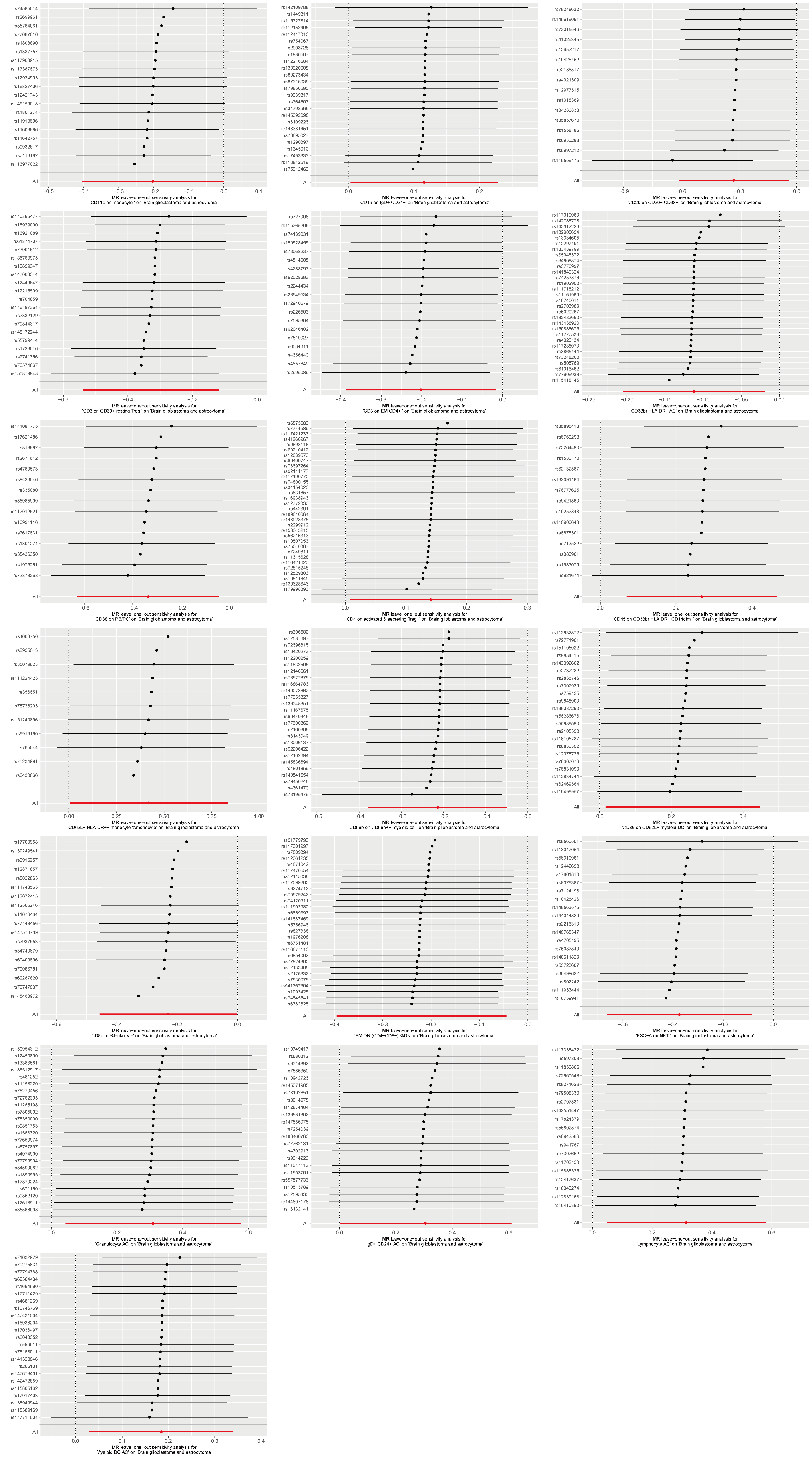


Supplementary Fig.4 We further validated the results through sensitivity analysis and found no evidence that any single SNP significantly influenced the overall causal relationship.
