# Supplementary material for: Mediation Mendelian randomization analysis of immune cell phenotypes and glioma risk: unveiling the regulation of cerebrospinal fluid metabolites
Source: Discov Oncol. 2025 May 9;16:712. doi: 10.1007/s12672-025-02499-y (PMC12064550; doi:10.1007/s12672-025-02499-y)
Supplement: Supplementary file 5 — Additional file 5. [file 12672_2025_2499_MOESM5_ESM.docx]

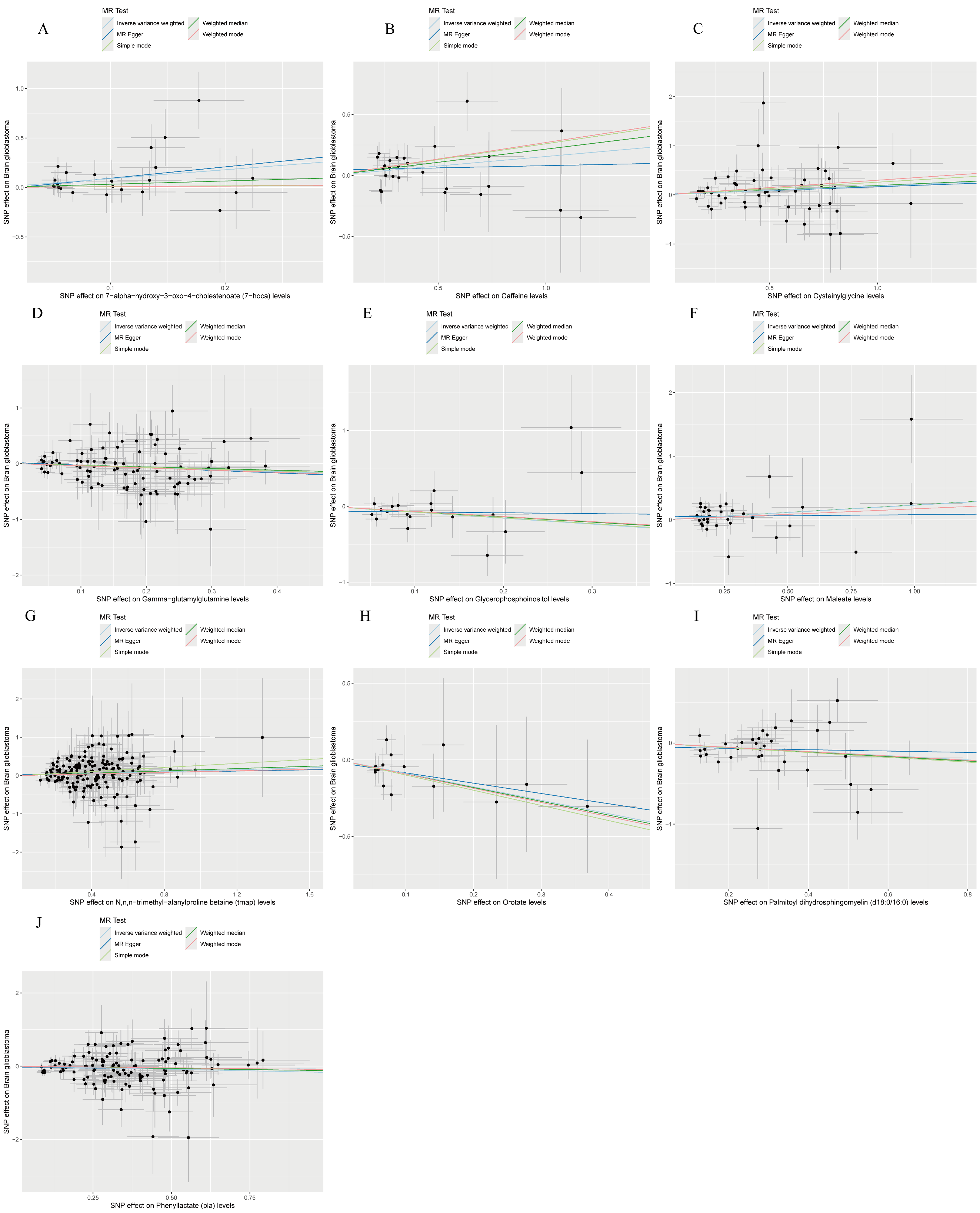


Supplementary Fig.5 Based on the results from the IVW method, we identified ten significant cerebrospinal fluid metabolites, such as 7-alpha-hydroxy-3-oxo-4-cholestenoate (7-hoca) levels, using a threshold of P < 0.05.
